# Mutational analysis of hepatitis E virus ORF1 "Y-domain": Effects on RNA replication and virion infectivity

**DOI:** 10.3748/wjg.v23.i4.590

**Published:** 2017-01-28

**Authors:** Mohammad Khalid Parvez

**Affiliations:** Mohammad Khalid Parvez, Department of Pharmacognosy, King Saud University College of Pharmacy, Riyadh 11451, Saudi Arabia

**Keywords:** Hepatitis E virus, Open reading frame 1, Y-domain, Palmitoylation, α-helix

## Abstract

**AIM:**

To investigate the role of non-structural open reading frame 1 “Y-domain” sequences in the hepatitis E virus (HEV) life cycle.

**METHODS:**

Sequences of human HEV Y-domain (amino acid sequences 216-442) and closely-related viruses were analyzed *in silico*. Site-directed mutagenesis of the Y-domain (HEV SAR55) was carried out and studied in the replicon-baculovirus-hepatoma cell model. *In vitro* transcribed mRNA (*pSK-GFP*) constructs were transfected into S10-3 cells and viral RNA replicating GFP-positive cells were scored by flow cytometry. Mutant virions’ infectivity was assayed on naïve HepG2/C3A cells.

**RESULTS:**

*In silico* analysis identified a potential palmitoylation-site (C_336_C_337_) and an α-helix segment (L_410_Y_411_S_412_W_413_L_414_F_415_E_416_) in the HEV Y-domain. Molecular characterization of C_336_A, C_337_A and W_413_A mutants of the three universally conserved residues showed non-viability. Further, of the 10 consecutive saturation mutants covering the entire Y-domain nucleotide sequences (nts 650-1339), three constructs (nts 788-994) severely affected virus replication. This revealed the indispensability of the internal sequences but not of the up- or downstream sequences at the transcriptional level. Interestingly, the three mutated residues corresponded to the downstream codons that tolerated saturation mutation, indicating their post-translational functional/structural essentiality. In addition, RNA secondary structure prediction revealed formation of stable hairpins (nts 788-994) where saturation mutation drastically inhibited virion infectivity.

**CONCLUSION:**

This is the first demonstration of the critical role of Y-domain sequences in HEV life cycle, which may involve gene regulation and/or membrane binding in intracellular replication complexes.

**Core tip:** The function of hepatitis E virus (HEV) Y-domain remains elusive. *In silico* analysis of closely-related virus sequences mapped a potential palmitoylation-site (CC) and α-helix segment (LYSWLFE) in the Y-domain. Mutant replicons of the universally conserved residues C_336_, C_337_ and W_413_ showed non-viability. Saturation mutations in the Y-domain (nucleotide sequences 788-994) severely affected RNA replication, revealing their post-transcriptional indispensability. Notably, the three residues corresponded to the non-conserved codons, indicating their post-translational essentiality. RNA secondary structure prediction showed hairpin formations by the critical bases (788-994), where mutations drastically affected virion infectivity. This is the first demonstration of the critical role of Y-domain sequences in HEV life cycle that warrants further molecular/biochemical studies.

## INTRODUCTION

The eukaryotic positive single-strand (+ss) RNA viruses share evolutionarily-conserved functional and putative domains, and even amino acid (aa) sequences in their nonstructural/replicase polyproteins[1]. In infected cells, one of the polyprotein proteolytic products, the methyltransferase (MTase), catalyzes 5’ capping of viral mRNA and interacts with cytoplasmic membranes, essential for establishing replication complexes[2]. In addition to the MTase-domain, studies have also shown sequence and structural conservation of the downstream Y-domain in viral polyproteins[1,3,4]. Recently, sequence analysis of human, animal and plant viruses of the alphavirus-like superfamily has suggested the Y-domain as an extension (“iceberg” region) of the MTase C-terminal (“core” region), and identified its homolog in nodaviruses[5]. Further, universally conserved cysteine residues have been identified in the core region of animal viruses, such as Semliki Forest virus (SFV) and Sindbis virus (SINV), and closely-related plant viruses, including bromo mosaic virus (BMV), bamboo mosaic virus (BaMV), alfalfa mosaic virus (AMV), tomato mosaic virus (ToMV), tobacco mosaic virus (TMV) and cucumber mosaic virus (CMV), critical for RNA capping and replication[6-13].

Moreover, mutational analysis of SFV-nonstructural protein 1 (nsP1) has shown indispensability of both the core and Y-domain residues for RNA capping activity[14,15]. In SINV-nsP1, deletions of core residues (aa 442-492) not only abolished MTase activity but also virus infectivity[16]. Notably, the capping was completely retained by truncated SINV-nsP1 (aa 1-448)[17] and BaMV-replicase (aa 1-442)[7], which ended only 30-40 residues down in the Y-domain. The MTase-domain (N-terminal) alone, therefore, seems insufficient for viral 5’ mRNA capping that is actually complemented by the combined sequences of core and Y. Thus, it is the “core-Y” region that undergoes post-translational palmitoylation, required for membrane binding through an amphipathic α-helix to form replication complexes on cytoplasmic membranes[5,18-21].

**Figure 1:**
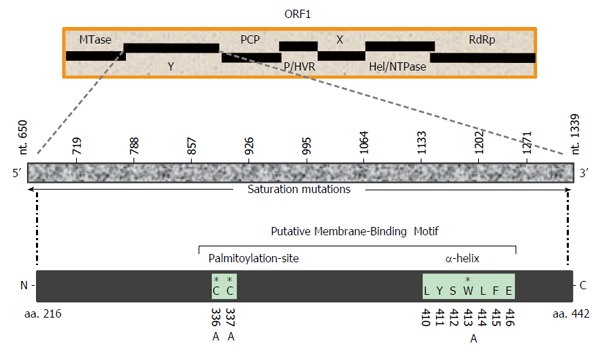
Schematic representation of hepatitis E virus nonstructural polyprotein (ORF1) domain organization, showing the undefined Y-domain. Saturation mutations covering the entire Y-domain (nts 650-1339; 10 constructs of 68 bases each) as well as specific amino acid (C_336_, C_337_ and W_413_) mutations within the predicted membrane-binding motif are shown. MTase: Methyltransferase; Y: Undefined; PCP: Papin-like cysteine protease; P/HVR: Proline-rich/hypervariable region; X: Macro; Hel/NTPase: Helicase/nucleotide triphosphatase; RdRp: RNA-dependent RNA polymerase.

The hepatitis E virus (HEV), the only Hepevirus of the alphavirus-like superfamily, is the etiological agent of acute and chronic hepatitis E in humans[22,23]. The HEV +ssRNA genome (about 7.2 kb) contains three partially overlapping open reading frames (ORFs): ORF1, ORF2 and ORF3, flanked by 5’ and 3’ short untranslated-regions[24,25]. Of these, the largest gene, ORF1 (5109 bases), encodes the nonstructural polyprotein (1703 residues) essential for viral RNA replication in infected cells[26-28]. Homologous to the alphavirus polyprotein structural organization, the MTase-domain is followed by the Y-domain in HEV ORF1 (Figure 1). While the 5’ mRNA capping activity of the ORF1 MTase-domain (N-terminal) is well characterized and implicated in RNA replication[29,30], the function of the Y-domain remains completely unexplored. Therefore, the present study was postulated to investigate a potential role of Y-domain sequences in HEV life cycle, using the replicon-baculovirus-hepatoma cell model.

## MATERIALS AND METHODS

### In silico analysis

The Y-domain sequences of human HEV strains (GenBank; *n* = 206), belonging to the four genotypes (HEV1, HEV2, HEV3 and HEV4) as well those of closely-related +ssRNA viruses were analyzed using the online bioinformatics tools *Multalin* (http://multalin.toulouse.inra.fr/multalin/cgi-bin/multalin.pl) and *ClustalW* 1.8 (http://embnet.vital-it.ch/software/ClustalW.html). Prediction of peptide secondary structure/amphipathic helix was done by *PSIPRED* (http://bioinf.cs.ucl.ac.uk/psipred) and *PROFsec* (http://bioinf.cs.ucl.ac.uk/psipred). The program *RNAstructure* (http://rna.urmc.rochester.edu/RNAstructureWeb/index.html) was used to predict RNA secondary structures.

### Construction of Y-domain mutant replicons

Mutations were introduced in HEV1-SAR55 (accession no. AF444002) full-length genomic replicon (*pSK-GFP*; a kind gift from Dr Suzanne Emerson, National Institutes of Health, Bethesda, MD, United States) by site-directed mutagenesis as described elsewhere[27]. Ten consecutive saturation mutants that covered the entire Y-domain nucleotide sequences (nts 650-1339), including *pSK-GFP-Ydom1* (nts 650-718), *pSK-GFP-Ydom2* (nts 719-787), *pSK-GFP-Ydom3* (nts 788-856), *pSK-GFP-Ydom4* (nts 857-925), *pSK-GFP-Ydom5* (nts 926-994), *pSK-GFP-Ydom6* (nts 995-1063), *pSK-GFP-Ydom7* (nts 1064-1132), *pSK-GFP-Ydom8* (nts 1133-1201), *pSK-GFP-Ydom9* (nts 1202-1270) and *pSK-GFP-Ydom10* (nts 1271-1339), were constructed by changing every possible nucleotide without altering the aa sequences. In addition, three aa mutants, including *pSK-GFP-YdomC336A*, *pSK-GFP-YdomC337A* and *pSK-GFP-YdomW413A*, of universally conserved residues within the predicted membrane binding motif were constructed. Replicon constructs *pSK-GFP-WT* and *pSK-GFP-G816V*[27] served as positive and negative controls, respectively.

Briefly, polymerase chain reaction (PCR) was carried out in a 50 μL reaction volume with appropriate amounts of *pSK-GFP* plasmid, forward and reverse primers, dNTP mix, DNA polymerase and buffer, under thermal conditions as per the manufacturer’s instructions (TaKaRa Bio Inc, Shiga, Japan). The PCR products were gel electrophoresed to confirm full amplification, *Dpn*I (Invitrogen, Carlsbad, CA, United States) digested to eliminate residual template, and transformed into XL-blue DH5α competent cells (Stratagene, San Diego, CA, United States) by heat-shock method. Transformed colonies were selected on ampicillin-agar plates, and isolated DNA (Plasmid Mini-prep Kit; Qiagen, Hilden, Germany) were screened by restriction-digestion. Mutant constructs were confirmed by sequencing (Invitrogen) and stock DNAs were prepared (Plasmid Midi-prep Kit; Qiagen) for *in vitro* transcription and transfection experiments.

### Human hepatoma cell culture

The S10-3 and HepG2/C3A cells, derivatives of human hepatoma lines HuH7 and HepG2, respectively (kind gifts of Dr Suzanne Emerson, NIH) were maintained in DMEM-GlutaMax (Invitrogen) supplemented with 10% heat-inactivated bovine serum (Gibco, Thermo Fisher Scientific, Waltham, MA, United States) and 1x penicillin-streptomycin mix (Gibco) at 37 °C with 5% CO_2_ supply[31]. For experimental studies, cells were seeded in 12-well (1.0 × 10^6^ cells/well) or 24-well (0.5 × 10^6^ cells/well) flat-bottom culture plates.

### In vitro capped mRNA synthesis and cell transfection

The replicon constructs (cDNA) were linearized with *Bgl*II (Invitrogen) and *in vitro* transcribed in the presence of anti-reverse cap analog (ARCA; Ambion, Austin, TX, United States) essentially as described elsewhere[31]. The transcription mix was gel electrophoresed to check the size, integrity and quality of the capped-mRNA, followed by transfection into S10-3 cells. The transfected cultures were incubated at 34.5 °C for 6 d to allow for optimal production of green fluorescent protein (GFP), indicating virus replication. S10-3 culture transfected with *pSK-GFP-WT* transcript showing green fluorescence served as positive control, while that receiving *pSK-GFP-G816V*, a lethal mutant[27], served as negative control. All transfections were performed in duplicate and repeated for reproducibility.

### RNA trans-encapsidation and virion infectivity assay

The mutant RNA encapsidation into viral ORF2 (capsid) proteins over-expressed by a recombinant baculovirus (vBacORF2) that could produce virus particles in S10-3 cells was performed, and tested for their infectivity on naïve HepG2/C3A cells[32,33]. In sum, RNA transfected S10-3 cells (in 12-well plates) were transduced with vBacORF2 on the following day. On day 6 post-transfection (*i.e*., day 5 post-transduction), lysates were prepared by vigorous vortexing of the cells in 10 × PBS, and normalized with sterile water. Lysates (inoculums) were cleared by centrifugation and overlaid on the HepG2/C3A cells (in 24-well plates), following 2.5 h incubation at 37 °C. The inoculums were aspirated and complete medium was added, and the cells were incubated for 6 d to establish infection and GFP production. The assay was done in duplicate and repeated for reproducibility.

### Fluorescence microscopy

The replication fitness of the mutant RNA was indirectly assessed by careful observation of GFP-positive S10-3 and HepG2/C3A cells under fluorescence microscope. The expression of capsid protein in S10-3 cells was confirmed on day 4 post-transduction[32]. Briefly, vBac-ORF2 transduced cells (in 8-chambered glass slides) were immune-stained with anti-ORF2 chimp sera and Alexa Fluor 488 goat anti-human IgG (Molecular Probes, Eugene, OR, United States). Following mounting with Vectashield (Vector Laboratories, Burlingame, CA, United States), slides were observed under FITC filter-aided indirect fluorescence microscope (H600L; Nikon, Tokyo, Japan).

### Flow cytometry

On day 6, the duplicated transfected S10-3 cultures (24-well plates) were harvested by trypsinization[32]. In sum, each culture suspension (about 500 μL in cold PBS) was cleared at 4 °C and cell pellets were re-suspended in 300 μL of cold PBS. The samples were immediately subjected to flow cytometry (10000 cells counted/sample), and data was analyzed for GFP-positive cells.

## RESULTS

### Mapping of potential palmitoylation-site and α-helix segment in the Y-domain

**Figure 2:**
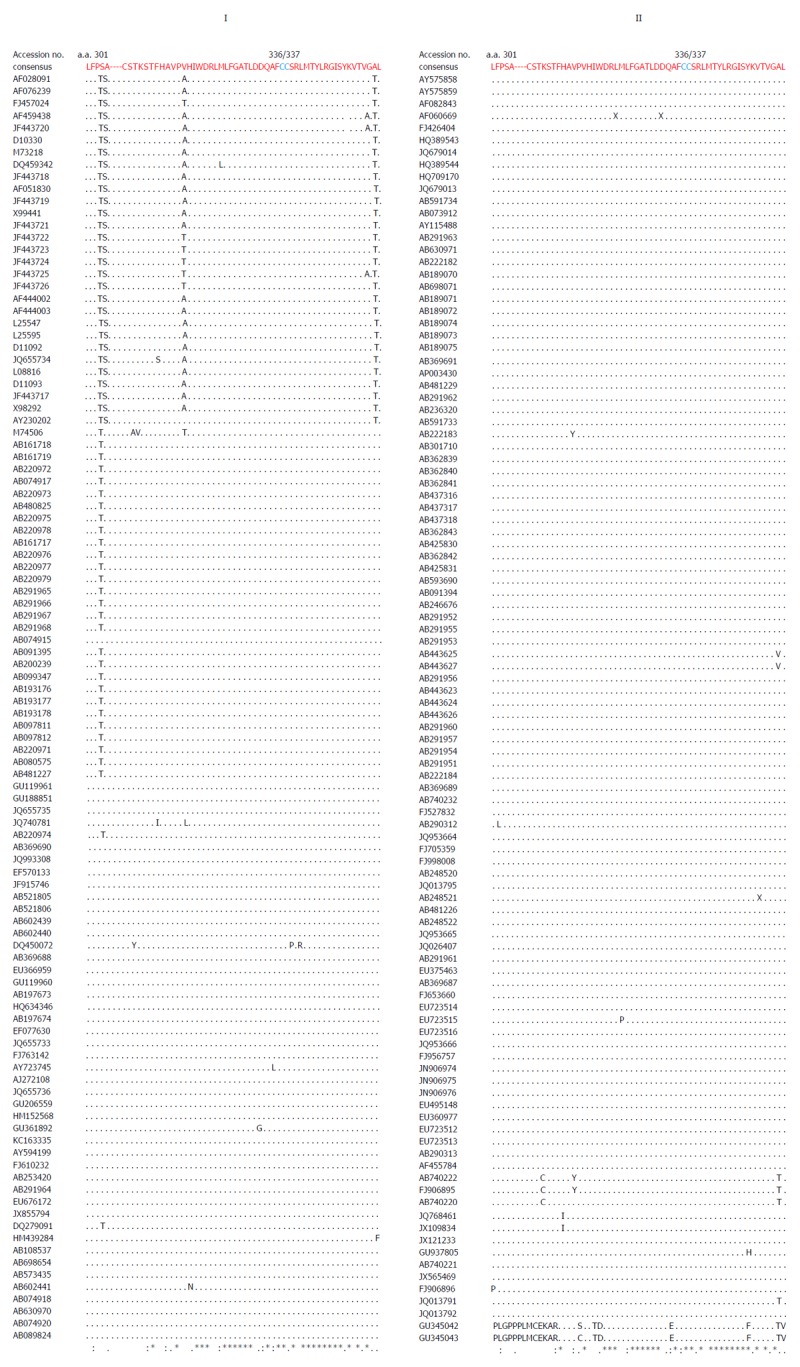
Multiple sequence analysis of ORF1 Y-domain of hepatitis E virus strains (GenBank; *n* = 206) showing the conservation of predicted palmitoylation-site residues (C_336_C_337_).

**Figure 3:**
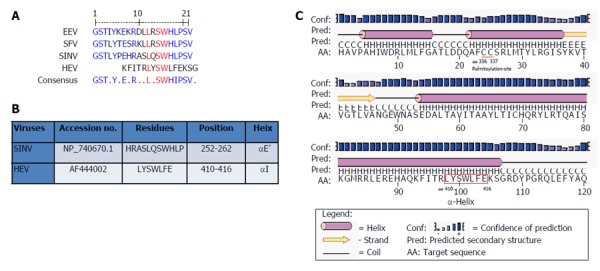
*In silico* analysis of Y-domain of hepatitis E virus and closely-related viruses. A: Residue and positional conservation of L_310_, S_312_ and W_313_ (highlighted in red) in the predicted membrane-binding α-helix among HEV, EEV, SFV and SINV; B: Predicted HEV α-helix LYSWLFE counterpart of SINV; C: Secondary structure prediction of α-helix in the HEV Y-domain.

**Figure 4:**
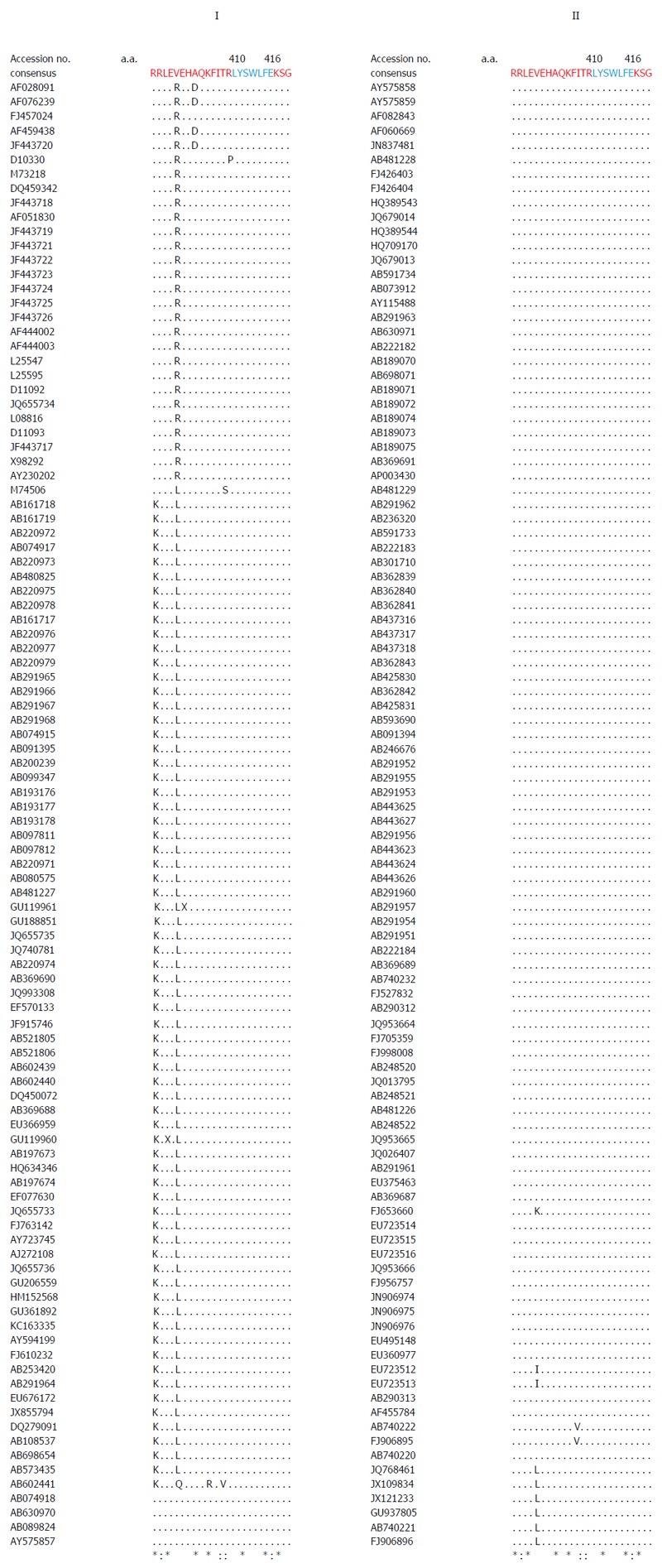
Multiple sequence analysis of human hepatitis E virus strains (GenBank; *n* = 206), showing the highly conserved segment (L_310_Y_311_S_312_W_313_L_314_F_315_E_316_) of predicted membrane-binding helix (α1) within the ORF1 Y-domain.

Multiple sequence analysis of HEV strains and representative alphaviruses identified a potential palmitoylation-site homolog CC and an α-helix counterpart LYSWLFE in the Y-domain, predicted for cytoplasmic membrane binding. The residues C_336_C_337_ and their positions were highly conserved across the available sequences of all four human HEV genotypes (Figure 2). In the predicted α-helix, while residue and positional conservation of L_410_, S_412_ and W_413_ were found among HEV and SFV, SINV and equine encephalitis virus (EEV) (Figure 3), the segment L_410_Y_411_S_412_W_413_L_414_F_415_E_416_ showed a high conservation within the HEV genotypes (Figure 4).

### Y-domain nts 788-994 are indispensable for virus replication

**Figure 5:**
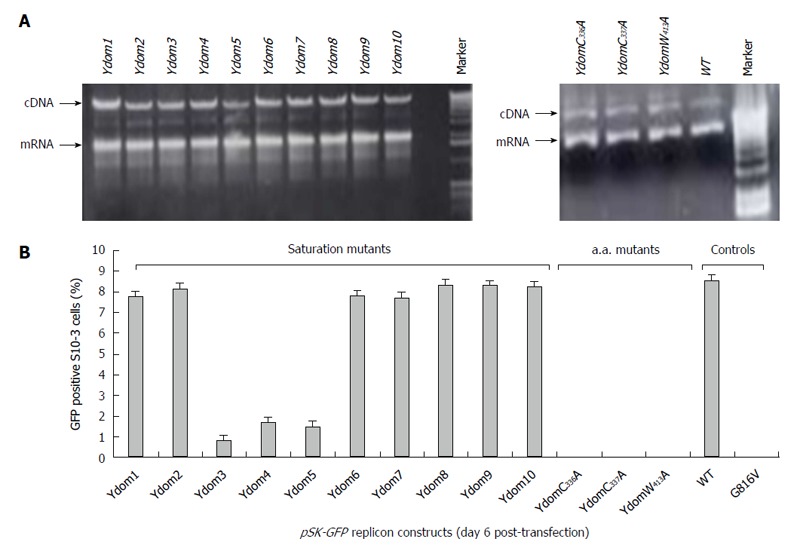
Molecular characterization of hepatitis E virus Y-domain sequences. A: Agarose-gel electropherograms showing the gross RNA yield of *pSK-GFP* saturation mutants: *Ydom1* to *Ydom10* (left panel) and specific amino acid mutants: *YdomC_336_A*, *YdomC_337_A* and *YdomW_413_A* (right panel), compared to wild-type (*WT*); B: Flow cytometry analysis of GFP-positive S10-3 cells, showing the replication competence of the Y-domain mutant replicons.

The saturation mutations introduced in the cDNA did not affect the gross yield of *in vitro* synthesized transcripts (Figure 5A, left). Of the 10 consecutive mutant transcripts (*pSK-GFP-Ydom1* to *Ydom10*), mutants of nts 788-994 (*Ydom3, 4* and *5*) drastically affected RNA replication by > 92% in S10-3 cells, whereas those of nts 650-787 (*Ydom1* and *2*) and nts 995-1339 (*Ydom6, 7, 8, 9* and *10*) had very mild or insignificant effect on viability compared to the wild-type (*Ydom-WT*) (Figure 5B). This clearly demonstrated the indispensability of the internal sequences but not the up- or downstream sequences of the Y-domain at transcriptional level.

### Universally conserved C_336_, C_337_ and W_413_ are critical for RNA replication

Similar to the saturation mutations, aa substitutions in the cDNA had no effect on the gross yield of *in vitro* synthesized RNA (Figure 5A, right). Introduction of C_336_A, C_337_A and W_413_A substitutions within the predicted membrane binding motif of Y-domain completely abolished virus replication (Figure 5B). Interestingly, the aa C_336_, C_337_ and W_413_ corresponded to codons (nts 1031-1033, 1034-1036 and 1213-1215, respectively) that were shown to be dispensable by saturation mutations. This very clearly indicated their post-translational functional/structural essentiality in virus replication, probably through membrane binding in intracellular replication complexes.

### Effects of RNA hairpin/stem-loop structures (nts 788-994) on virion infectivity

**Figure 6:**
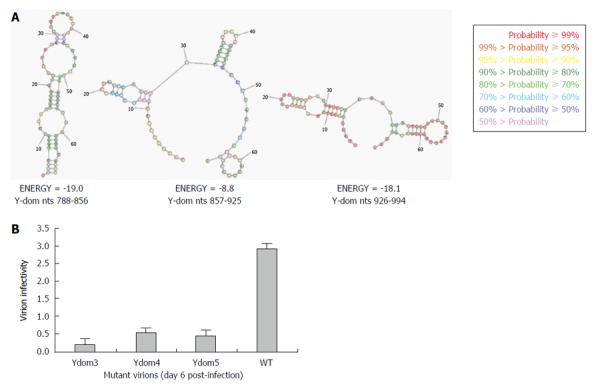
Analysis of Y-domain mutant virions’ infectivity. A: *In silico* prediction of stable RNA hairpin/stem-loop structures (wild-type) of three consecutive regions (*Ydom3*: nts 788-856, *Ydom4*: nts 857-925 and *Ydom5*: nts 926-994); B: Flow cytometry analysis of naïve HepG2/C3A cell infectivity by trans-encapsidated virions harboring the three saturation mutant RNAs (*Ydom3*, *Ydom4* and *Ydom5*).

As revealed by transfection results, *Ydom3* (nts 788-856) had the most drastic effect on RNA replication, followed by *Ydom5* (nts 926-994) and *Ydom4* (nts 857-925). In line with this, while nts 788-856 formed the most stable RNA hairpin/stem-loop compared to nts 926-994, nts 857-925 presented the least stable structure (Figure 6A). This strongly supported the deleterious effects of saturation mutations that could completely unzip and destabilize the RNA secondary structures, critical for virus replication. Further, the three saturation mutants (*Ydom3, 4* and *5*) with destabilized hairpins were assessed for RNA trans-encapsidation and virion infectivity. All the three RNA mutants showed high inhibitory effects on virion infectivity, where *Ydom5* (nts 788-856) had the most drastic effect compared to *Ydom4* (nts 857-925) and *Ydom5* (nts 926-994) (Figure 6B). This strongly supported the deleterious effects of saturation mutations that could completely unzip and destabilize the RNA secondary structures, critical for virus replication and infectivity.

## DISCUSSION

The alphavirus-like superfamily includes pathogenic animal viruses like HEV, SFV and SNV, and plant viruses such as BMV, AMV, ToMV, TMV and CMV, having +ssRNA genomes. In infected cells, viral nonstructural/replicase proteins recruit and catalyze 5’ capping of their genomic RNA onto cytoplasmic membranes, and synthesize the complementary strand that is sequestered in membrane-bound replication complexes[34]. In the present mutational study, the role of the ORF1 Y-domain was investigated, using an HEV replicon that expressed GFP (instead of the ORF2 protein) as a reporter of virus RNA replication and infectivity in hepatoma cells. Of the series of replicon constructs with saturation mutations in the Y-domain, the nts 788-994 mutants drastically affected RNA replication and particle infectivity, showing the critical role of the Y-domain internal sequences in the HEV life cycle.

A universal feature of +ssRNA viruses is the cytoplasmic membrane binding of nonstructural proteins that requires post-translational fatty acylation. Palmitoylation of cysteine residues has been widely studied in a number of cellular and viral proteins[35]. In eukaryotic α-subunit of G[36], Ras[37], and neuronal GAP-43[38] membrane binding proteins, a single cysteine in the only identified motif (MGC) undergoes palmitoylation. Similarly, in alphavirus-like superfamily nonstructural/replicase proteins, at least one cysteine is found in the homologous palmitoylation-sites. In SFV, the tight membrane association of nsP1 was shown due to increased palmitoylation of one of the highly conserved cysteines (C_418_C_419_C_420)_) within the MTase core region, where C→A changes completely abolished palmitoylation[15,39]. These mutations were also shown to attenuate virus replication and pathogenicity in infected mice[8,9]. Likewise, the C_420_A mutation in SNV-nsP1 also had inhibitory effect on virus replication[8]. In ToMV, the palmitoylation-site counterpart contains three conserved but distantly located cysteines (C_179_/C_186_/C_581_), wherein C→S substitution strongly decreased membrane binding, 5’ capping and RNA replication[10]. Akin to this, mutating the two conserved cysteines (C_179_/C_186_) at the same position aborted replication of BMV[11] and AMV[12]. Interestingly, mutation of the only C_461_ of CMV-1a also abrogated membrane binding and RNA replication[13]. In accordance with this, C→A substitution of the highly conserved C_336_C_337_ residues in the predicted palmitoylation-site of HEV Y-domain also abolished RNA replication completely.

Moreover, in addition to cysteine palmitoylation, many of the viral polyproteins contain consensus hydrophobic sequences for tight membrane binding[40]. In SFV-nsP1, the amphipathic segment GSTLYTESRKLLRSWHLPSV (aa 245-264) that forms an α-helix, has been implicated in membrane binding[18,19] and RNA replication[21]. A mutational analysis of BMV and CMV-1a, wherein virus replication was abolished while affecting membrane binding and RNA recruitment, suggested the structural conservation of its amphipathic helix A[13]. Similarly, the poliovirus-2C[41] and hepatitis C virus (HCV)-NS5A[42] have been implicated in amphipathic helix-modulated interactions with intracellular membranes. In this report, a highly conserved segment LYSWLFE (aa 410-416) was mapped as the α-helix counterpart of the ORF1 Y-domain. Sequence alignment showed the universal conservation of L_410_, S_412_ and W_413_ among HEV and the alphavirus-like superfamily. In line with this and the previously reported deleterious effect of W_259_A change on SFV replication[21], the specifically selected W_413_A completely abolished HEV RNA replication. Notably, tryptophan is a signature hydrophobic residue that is critical for α-helical protein folding for protein-protein interactions. Of the several examples, W_630_ in the conserved motif KTXXXW of amphipathic helix of the G protein-coupled receptor Frizzled (C-terminal) has been shown crucial in intracellular protein interactions[43]. Such functional/structural homology suggests the essentiality of the Y-domain (C-terminal) predicted α-helix in HEV replication that may embody common principles of viral nonstructural proteins in membrane interaction.

In conclusion, the present study shows the indispensability of highly conserved sequences (nts 788-994) of ORF1 Y-domain in HEV RNA replication and infectivity. Also, the universally conserved C_336_, C_337_ and W_413_ residues corresponding to the dispensable codons within the predicted membrane binding motif of Y-domain are critical for virus viability. Taken together, this is the first demonstration of the essentiality of Y-domain in the HEV life cycle, probably through gene regulation and/or membrane binding in replication complexes. Nevertheless, further molecular and biochemical studies are recommended to validate these findings.

## COMMENTS

### Background

Hepatitis E virus (HEV) is the etiological agent of acute and chronic hepatitis in humans, worldwide. While there has been great progress in understanding the virus biology, the function of nonstructural open reading frame 1 “Y-domain” remains completely unexplored.

### Research frontiers

The author has performed *in silico* analysis of closely related single-strand RNA virus sequences and mapped a potential palmitoylation-site and α-helix segment in the HEV nonstructural Y-domain. Mutational characterization of the viral replicon in S10-3 cells has shown the criticality of “Y-domain” residues C_336_C_337_ and W_413_ of the putative palmitoylation and helix, respectively. Further introduction of base mutations in the “Y-domain” severely affected RNA replication, revealing their post-transcriptional essentiality. Notably, the universally conserved C_336_C_337_ and W_413_ corresponded to non-conserved codons, indicating their post-translational indispensability. Moreover, RNA secondary structure prediction showed hairpin formations by the critical bases (nts 788-994) where mutations drastically affected virion infectivity of naïve HepG2 cells.

### Innovations and breakthroughs

This is a novel study showing a critical role of the hitherto undefined Y-domain in HEV genomic RNA replication and infectious virion production in cultured liver cells.

### Applications

The data warrants further biochemical and molecular studies of the Y-domain towards understanding HEV replication.

### Terminology

The putative palmitoylation and α-helix motifs of the HEV Y-domain may be key to intracellular membrane-binding, essential for RNA replication and production of infectious virions.

### Peer-review

The author showed a critical role of the Y-domain in HEV genomic RNA replication and infectious virion production.
